# K-562 Extracellular Vesicles Partially Protect Intact Cells from Oxidative Stress and Provide Limited Resistance to Imatinib

**DOI:** 10.3390/cimb47080666

**Published:** 2025-08-18

**Authors:** Jiana Sbiet, Einat Beery, Zinab Sarsor, Pia Raanani, Orit Uziel

**Affiliations:** 1 The Felsenstein Medical Research Center, Rabin Medical Center, Petah-Tikva 4941492, Israel; 2 Institute of Hematology, Davidoff Cancer Center, Petah Tikva 4941492, Israel; 3 The Gray Faculty of Medical & Health Sciences, Tel-Aviv University, Ramat-Aviv, Tel Aviv 6997801, Israel

**Keywords:** chronic myeloid leukemia, oxidative stress, proliferative stress, EVs, reactive oxygen species, imatinib mesylate

## Abstract

Chronic myeloid leukemia (CML) results from the formation of the BCR-ABL1 chimeric protein which serves as a target for clinically used tyrosine kinase inhibitors (TKIs), such as imatinib mesylate (IM). Although very efficient, the development of resistance to TKIs remains a critical issue for a subset of patients. In our study we aimed to identify one aspect of IM resistance in K-562 cells, a cell line used as a model for CML. Secreted from all cell types, extracellular vesicles (EVs) are nanoparticles that function as mediators of cell–cell communication. Upon engulfment by other cells they may modulate their phenotype. IM is linked to changes in oxidative metabolism in K-562 cells. Our study explored the putative involvement of EVs secreted from K-562 cells in providing protection from oxidative stress and resistance to IM in these cells. The results of our study showed that the protection from oxidative stress provided by previously exposed K-562 cell, derived EVs is only partial. Similarly, these EVs provided intact K-562 cells with some resistance to IM treatment. These results may suggest that resistance to IM may develop and expand to other cells by EVs that are secreted from already resistant cells, similar to a horizontal transfer of resistance provided by plasmids in bacteria.

## 1. Introduction

Chronic myeloid leukemia (CML) comprises ~15% of all newly diagnosed leukemias in adults. The Bcr-Abl1 fusion gene encoding a tyrosine kinase that typifies CML, leads to an uncontrolled proliferation and inhibition of apoptosis in progenitor cells within the bone marrow. This chimeric protein is a part of the newly formed Philadelphia chromosome, expressed as a fusion between the Abelson murine viral leukemia oncogene homolog 1 (*Abl1*) tyrosine kinase gene at chromosome 9 with the break point cluster region (*Bcr*) gene at chromosome 22. The resulting chimeric oncogene is a constitutively active Bcr-Abl tyrosine kinase [1].

Oxidative stress is among the downstream signaling pathways promoted by BCR-ABL1 [2].

Imatinib mesylate (IM), the first tyrosine kinase inhibitor (TKI) approved for CML, blocks the activity of the BCR-ABL1 tyrosine kinase by hindering its ATP-binding site and consequently induces apoptosis of CML cells [3]. Cells exposed to IM undergo rapid apoptosis, which in most patients manifests as a complete hematologic response. However, numerous leukemic cells from the hematopoietic stem cell population are resistant to IM therapy and their survival does not seem to be dependent solely on BCR-ABL1 activity [4]. Recent studies point to multiple factors that can contribute to the development of resistance to TKIs including IM [5]. IM resistance may stem from over expression or amplification of the Bcr-Abl gene and point mutations within the Bcr-Abl kinase domain that interfere with imatinib binding. Additionally, alterations in drug influx and efflux or activation of Bcr-Abl independent pathways may lead to IM resistance [5].

Oxidative stress has been linked to CML in several levels. BCR-ABL1 induces the production of reactive oxygen species (ROS) in hematopoietic cells leading to oxidative stress which is involved in its anti-apoptotic effects. As such, oxidative stress is harmful to patients with CML. On the other hand, oxidative stress damages DNA and interferes with DNA repair, leading to genomic instability, which may promote apoptosis [6,7,8] and, therefore, may be considered beneficial to CML patients. The status of oxidative stress in CML was also linked to the resistance of the neoplastic cells to IM and its derivatives [9], which may support a negative effect in CML patients.

Secreted by practically all cell types, extracellular vesicles (EVs) differ in their biogenesis, release pathways, size, content, and function. The three main subtypes of EVs are apoptotic bodies, microvesicles, and exosomes. Originated from early endosomes and processed into multivesicular bodies, exosomes are 30–150 nm in diameter. During the development of leukemia, leukemic cell-derived exosomes are implicated in the transformation of the normal hematopoietic niche toward a malignant tumor-supportive microenvironment which contributes to the progression of the malignancy as well as the drug resistance [10]. A number of recent studies have revealed that exosomes or EVs are secreted by CML cells and demonstrated their capability to induce the formation of new vessels, suggesting their role in angiogenesis in the bone marrow of patients with CML [11,12]. These include micro RNA such as miR-214, miR29a, miR01, miR126, and miR-320; long noncoding RNA including MALAT1, MANTIS and proteins such as NFkB and STAT3 [13].

We have planned the described study in light of the need to deepen our understanding about development of resistance to IM in patients with CML and the increasing knowledge about exosomes and their contribution to drug resistance. For this purpose, we have studied the CML model cell line, K-562, in our study. We aimed at deciphering the putative role of EVs in conferring IM resistance to CML, in the presence of oxidative stress using a CML cell line model.

## 2. Materials and Methods

### 2.1. Cell Lines

CML cell line, s-K562 (Catalog number CCL-243), expressing the E14a2 variant of BCR-ABL1, sensitive to IM, was cultured in RPMI-1640 medium (biological industries) supplemented with 10% fetal bovine serum (FBS), 1% L-glutamine and 1% penicillin-streptomycin (Biological Industries, Beit Haemek, Israel). Cells were grown in a 37 °C incubator in the presence of 5% CO_2_ and 90% humidity for 72 h. CML human cell line, r-K562, resistant to IM, was purchased from the ATCC (ATCC CRL-3344, Manassas, VA, USA) and cultured in RPMI-1640 medium (biological industries) supplemented with 10% FBS, 1% L-glutamine and 1% penicillin-streptomycin (Biological Industries, Beit Haemek, Israel) and 1–2 μM IM.

### 2.2. EVs Isolation

A total of 2–4 × 10^8^ cells were grown in RPMI-1640 medium supplemented with 10% EVs free FBS, 1% L-glutamine and 1% penicillin-streptomycin (Biological Industries, Beit Haemek, Israel). At 24–72 h later, the growth media were collected and centrifuged for 10 min at 1000× *g* to remove contaminating cells. Then, the supernatant was further centrifuged for 10 min at 2000× *g* to pellet more cell debris. The supernatant was then centrifuged once more for 30 min at 10,000× *g* at 4 °C. Afterwards, the supernatant was filtered by a 0.22 μM filter and then ultracentrifuged for 2 h at 110,000× *g* at 4 °C. Subsequently, the supernatant was removed and the pellets were washed with PBS by ultracentrifugation for 2 h at 110,000× *g* at 4 °C. The upper liquid phase was removed and the pellets were immersed in 700 μL PBS and kept at −80 °C. To prepare EVs free FBS, FBS was centrifuged at the ultracentrifugation at 100,000× *g*, 4 °C for 18 h and the supernatant was collected.

### 2.3. Characterization of EVs

EVs were characterized by using Nano-Sight Tracking Analysis (NTA) and electron microscopy.

### 2.4. Nano-Sight Tracking Analysis (NTA)

EVs were quantified by NTA, according to the manufacturer’s instructions (Malvern Panalytical, Cambridge, UK). Briefly, samples were diluted 1:100 in particle-free PBS (Biological Industries, Beit Haemek, Israel) and analyzed under constant flow conditions at 25 °C. Camera level was set to 13, number of repeats: 5X60sec and dilution of samples was usually 1:100.

### 2.5. Transmission Electron Microscopy (TEM) Analysis

We loaded 3 μL of EVs extract on a glow discharged lacey grids (EmiTech K100 labtech, Sussex, UK) that were blotted and plunged into liquid ethane using a Gatan CP3 automated plunger. The grids were subsequently stored in liquid nitrogen until use. Frozen specimens (samples with EVs embedded in vitreous ice) were transferred to a Gatan 914 cryo-holder and maintained at temperatures below −176 °C inside the microscope. We inspected the samples with a Tecnai G2 microscope (Thermo Fisher, Waltham, MA, USA). This instrument has an acceleration voltage of 120 kV and is equipped with a cryobox decontaminator. Images were taken using Digital Micrograph with a Mulitiscan Camera model 794 (Gatan, Pleasanton, CA, USA).

### 2.6. Western Immunoblotting Analysis

Characterization of the presence of exosomal markers on the isolated EVs was determined by Western blotting using antibodies against well-characterized EV protein markers: TSG101, CD63, CD9, and calnexin. Protein concentration of the isolated EVs was determined by using the Pierce BCA Protein Assay Kit (Thermo Fisher Scientific, Waltham, MA, USA). A total of 50 μg of protein was subjected to 10% sodium dodecyl sulfate–polyacrylamide gel electrophoresis (SDS-PAGE) and transferred to a nitrocellulose membrane. After blocking the membrane from unspecific antibodies binding it was then hybridized for 16 h at 4 °C with antibodies against CD63, CD9 (1:1000, Santa Cruz Biotech, Dallas, TX, USA), anti-TSG101 and calnexin (1:500, Abcam, Waltham, MA, USA). On the following day, the membrane was subjected to fluorescent-labeled secondary antibodies after washing from access unbound antibodies. Visualization was performed using the Odyssey analysis software 6.0 (Odyssey IR imaging system; LI-COR, Lincoln, NE, USA).

### 2.7. Proliferation Assay

Proliferation was assessed by the Trypan blue exclusion staining (Biological Industries). Trypan blue was mixed in 1:10 ratio with treated suspended cells and counted by using Countess (automated cell counter, Invitrogen, Carlsbad, CA, USA). Live cells stayed unstained while dead cells assimilated the dye (total cell count was deduced from the live and dead cells). Each time 100–200 cells were counted twice, and the results were plotted accordingly. The relatively small standard error means reflect the accuracy of the method.

### 2.8. RNA Extraction

RNA extraction from cells was performed by using the EZ-RNA II Isolation Kit reagent (Biological Industries Beit Haemek, Israel) according to provided manual instructions. Briefly, the membrane of the cells was lysed with guanidine thiocyanate detergent solution, followed by organic extraction of phenol-chloroform and alcohol precipitation of the RNA. RNA concentrations and its quality were quantified by using the Nano-Drop spectrophotometer with the ND-1000 software and by the Qubit-2 device (Thermo Fisher Scientific, Waltham, MA, USA). Then, total RNA that was extracted from cells and EVs was reverse transcribed by using the High-Capacity cDNA Reverse Transcription Kit (Applied Biosystem, Foster City, CA, USA) according to the manufacturer’s instructions. Briefly, 1000 ng of template RNA was added to 10 μL reaction mixture containing dNTP’s, random primers, RNase inhibitor, reaction buffer and a reverse transcriptase (RT). The incubation steps included 10 min at 25 °C followed by 120 min at 37 °C and terminated by a heating step at 85 °C for 5 min. Q-RT-PCR was performed to measure the expression of the *NRF2* gene. Reactions were carried out by using the PCRbio Fast Blue Mix (PCR Biosystems, London, UK), FAM labelled primers were purchased from Thermo Fisher Scientific (Waltham, MA USA) and the reaction conditions were 95 °C for 2 min, followed by 40 cycles of 95 °C for 5 s, and 60 °C for 20 s. Reactions were run and analyzed in the StepOne device (Thermo Fisher Scientific).

### 2.9. Cell Viability

Assessment of cell viability was done by the WST-1 Cell Proliferation Reagent (Roche, Basel, Switzerland). Cells were seeded in a 96-well plate and incubated with the WST-1 reagent for 0.5–4 h. The activity of mitochondrial enzymes reflecting cells viability was followed by a colorimetric assay based on the enzymatic ability to cleave a formazan dye. The levels of the obtained color were quantified by using a scanning multi-well spectrophotometer 405 nm (“Sunrise” ELISA, Tecan Group AG, Salzburg, Austria) and the results were calculated relative to the control results.

### 2.10. Exposure of Cells to Proliferative Stresses

The presence of various FBS concentrations in the cells’ growth medium affects their growth rate. To mimic a higher growth rate, we exposed the cells to various FBS concentrations (2.5–20%) and assess their growth rate by the above-mentioned methods.

### 2.11. Exposure of the Cells to Oxidative Stress

Exposure to hydrogen peroxide was used to apply an oxidative damage/stress on the cells. We exposed our cells to various concentrations of H_2_O_2_ and selected the concentration that eliminated about 50% of the cells.

### 2.12. Evaluation of EVs Uptake

In order to follow EVs uptake by K562 cells, 1 × 10^6^ cells/mL per well were plated in a 24-well culture plate, resuspended in EVs depleted RPMI media and incubated with FM-1-43-labeled EVs in different concentrations and time periods. The FM-1-34 manufacturer’s protocol was performed as follows: 700 μL of exosomes were incubated with 100 μg of ready to use FM-1-43 dye for 10 min in the dark. Then, the mixture was washed with PBS by using ultracentrifugation for two hours at 110,000 RCF. The pellet was resuspended in 700 mL of PBS and kept until used under light protection conditions.

K562 cells without EVs served as control. To determine the efficiency of the EVs uptake by cells 24 h post EVs exposure, the cells were harvested and subjected to flow cytometry analysis. Validation that the dye remains stably associated with our EVs throughout the extended experimental timeframe was executed up to 96 h. The percentage of K562 cells that have taken up the EVs and were FM-1-43 labeled was evaluated by following the FL-2 channel. Potential artifacts from dye transfer to cellular membranes independent of EV internalization was monitored by analyzing a control sample with cells that were not exposed to EVs but only to the dye itself. To exclude nonviable cells from the analysis of flow cytometry, 7-AAD (7-Aminoactinomycin D) nucleic acid dye was used (Tonbo Biosciences, San Diego, CA, USA).

### 2.13. Apoptotic Cell Death Assay

Apoptotic cell death of CML cells that were exposed to H_2_O_2_ for different times periods (0, 6, 9, 12, 14 and 24 h), was evaluated by Annexin V and PI staining by using the MEBCYTO^®^ Apoptosis Kit (MBL, Nagoya, Japan) according to the manufacturer’s instructions.

Briefly, K562 cells in 6-well plates were cultured with H_2_O_2_ for 24 h and collected by centrifugation at 1000 rpm for 5 min and resuspended with 90 μL BB buffer (Binding Buffer), then cells were incubated with 10 μL Annexin V-FITC and 5 μL PI (Propidium Iodide) for 15 min at RT in dark. Fluorescent cells were analyzed by flow cytometry and the data were analyzed using Kaluza Acquisition Software version 2.1.

### 2.14. Cell Cycle Analysis by Flow Cytometry

The cell cycle status of the various samples was analyzed by flow cytometry. Cells were processed by standard methods using propidium iodide staining of the DNA. Briefly, after cells were cultured in 6-well culture plate and exposed to H_2_O_2_ for various times periods (0, 6, 9, 12, 14 and 24 h), the cells were washed by centrifugation at 1000 rpm for 5 min at 4 °C, resuspended in 5 mL cold PBS and centrifuged at 1000 rpm for 5 min at 4 °C. Cells were subsequently pelleted and fixed by adding 4.5 mL 70% cold ethanol dropwise to 0.5 mL cold PBS, with gentle vortexing. The cells were then incubated for >2 h at −20 °C. Cells were then washed by PBS and centrifuged at 1000 rpm for 5 min 4 °C. Staining with propidium iodide (P4170, Sigma Aldrich, St. Louis, MO, USA) was conducted just prior to flow cytometry. Cells were pelleted and resuspended in 0.5 mL PBS containing 0.025 mL of PI (50 μg/mL) in PBS and 5 μL of 100 mg/mL DNase-free RNase (Sigma Aldrich, St. Louis, MO, USA). Cells were then incubated at 37 °C for 15 min. Samples were analyzed on a Beckman-Coulter Epics XL-MCL apparatus. The parameters were adjusted for the measurement of single cells using the forward and side scatter plots. Data analysis was performed using Kaluza Acquisition Software (Beckman Coulter International SA, Nyon, Switzerland).

### 2.15. Measurement of Reactive Oxygen Species (ROS) by Flow Cytometry

ROS content of K562 cells were determined by using the 2′,7′-dichlorofluorescein diacetate assay (D6883-SIGMA). After 0, 6, 9, 12, 14 and 24 h of cells cultured with H_2_O_2_, cells were diluted with 10 μM DCFH-DA and incubated for 30 min at 37 °C in the dark. Then the cells were collected and washed twice with PBS and resuspended in 1 mL of PBS. ROS was detected by flow cytometry (FACS Calibur, BD, San Jose, CA, USA) according to the manufacturer’s protocol.

### 2.16. EVs and Cell Co-Cultivation

The K562 cells were co-cultured with either r-K562-derived or K562-EVs for 24 h in a 96-well plate at 37 °C, and then treated with IM (1, 2 μM) for 24 and 48 h. Cell viability was measured using the WST-1 assay as described previously. As negative controls we used cells without EVs and IM treatment.

### 2.17. Statistical Analysis

Data are presented as average + standard error mean (SEM) for all experiments involving two groups. All other results (described in the proliferative stress effects section, cell cycle analyses and apoptosis) were analyzed using ANOVA and Dunnet, using the SAS software version 9.4. Student’s *t*-tests were conducted using the SPSS software version 29.

Of note, no statistical analyses were conducted with the data collected from the measurements of the NRF2 transcripts, apoptosis, the uptake experiemts and exosomal counts from either resistants or sensitive cells. This is due to the low number of experimental repeats.

## 3. Results

### 3.1. Isolation and Characterization of K-562 Derived EVs

**Figure 1:**
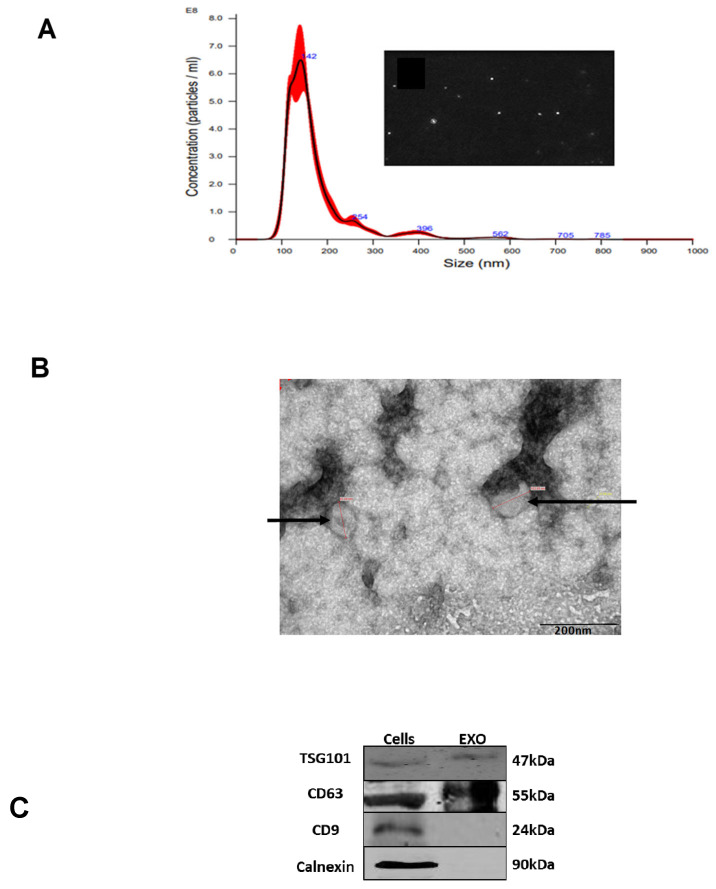
K-562 derived EVs. (**A**) K-562 cells were grown in EVs depleted medium for 72 h and EVs were isolated by ultracentrifugation. Shown is an example of a nanoparticle tracking analysis with highest concentration of particles ranging in size between 100 to 200 nm. The red area depicts the average of three experimental results exosomal isolation and shows the number of exosomes detected by the NTA. (**B**) Transition electron microscopy image of K-562 derived EVs depicted by the arrowheads. (**C**) Western immunoblotting analysis of K562 cells and their cognate EVs, showing the presence of two exosomal markers, TSG101 and CD63, and the absence of a non-exosomal marker, calnexin. Each experiment was conducted at least three times with biological replicates and technical replicates in each biological one.

To isolate EVs from the growth media of K-562 cells, we grew the cells for 72 h in EVs depleted media and collected the EVs from the conditional medium using differential ultracentrifugation. By nanoparticle tracking analysis (NTA), we identified the presence of a large amount of particles with an average size of 142 nm and by transmission electron microscopy (TEM) we have found vesicles in a similar size. Western immunoblotting analyses demonstrated the existence of two exosomal markers, TSG101 and CD63, and the absence of a non-exosomal protein, calnexin (Figure 1). Although these analyses may verify that the isolated EVs are exosomes, as we did not monitor exosomes known markers in this study, we define these vesicles as EVs.

### 3.2. Oxidative Stress Reduced Cell Proliferation and Viability and Increase EVs Secretion

**Figure 2:**
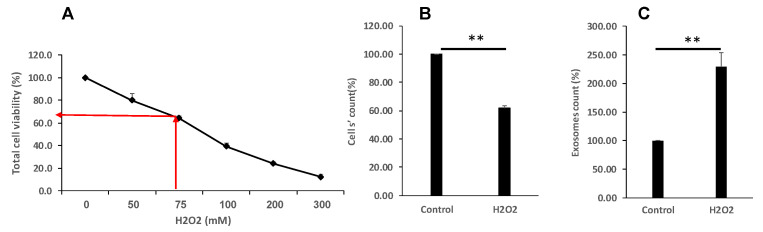
Oxidative stress differentially affects cell proliferation and EVs secretion. (**A**,**B**). K-562 cells were grown in the presence of various H_2_O_2_ concentrations and cells’ viability was assessed by the Trypan blue exclusion assay. The arrows depict the chosen concentration of H_2_O_2_ to which the cells were exposed to 75 μM. (**C**). Number of EVs released from cells grown in the presence of 75 μM H_2_O_2_, relative to EVs released from control intact cells. Each column represents mean ± standard error mean of three experiments. Each experiment was conducted three times with biological replicates and technical replicates in each one. ** *p* < 0.01, statistical significance as calculated by Student’s *t*-test.

To determine cell proliferation and viability after cell treatment with H_2_O_2_ we have stained the cells with Trypan blue. Exposure to H_2_O_2_ decreased cell numbers in a dose-dependent manner as shown in Figure 2A. An amount of 75 μM of H_2_O_2_ induced about a 30–40% reduction in cell viability and was chosen for further experiments throughout the study. Importantly, the number of EVs secreted from each cell increased more than twofold in response to the oxidative insult, as shown in Figure 2B.

### 3.3. Proliferative Stress Reduced Cell Proliferation and Viability and Increase EVs Secretion

**Figure 3:**
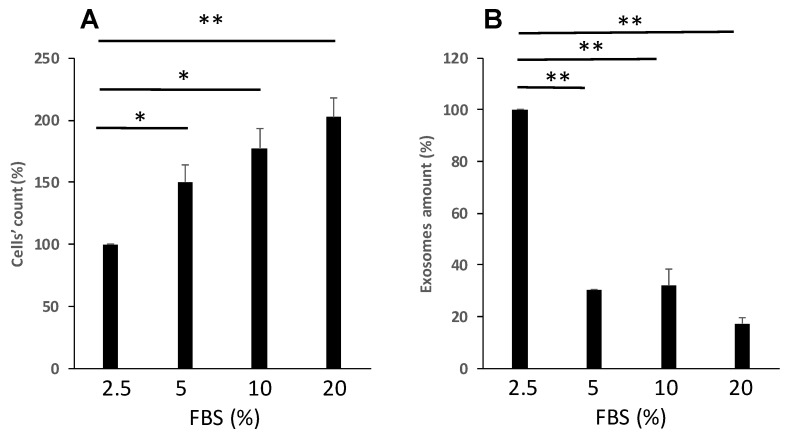
Proliferative stress differentially affects cell viability and secretion of EVs. K-562 cells were grown in the presence of increasing concentrations of FCS. (**A**). Cell viability was assessed by the Trypan blue exclusion assay. (**B**). Number of EVs released from cells grown in these conditions, relative to EVs released from control intact cells. Each column represents mean ± standard error mean of three experiments. Each experiment was conducted three times with biological replicates and technical replicates in each one. * *p* < 0.05, ** *p* < 0.01, ANOVA and Dunnet.

As another example of stress, we studied the effect of proliferative stress on the viability and EVs secretion. For this purpose, we grew the cells in the presence of reduced fetal calf serum (FCS), ranging between 20–2.5% FCS, and measure cells’ viability and number of secreted EVs. Of note, the 2.5% of FCS was chosen as the minimal concentration allowing cell growth. Figure 3 demonstrates that similar to the post oxidative stress results, the number of viable cells was reduced by half as the concentration of the media FCS dropped down. In parallel, number of secreted EVs increased fourfold when cells were starved for FCS.

### 3.4. Oxidative Stress Modulates the Amount of RNA Content and Nrf2 Levels of Evs

**Figure 4:**
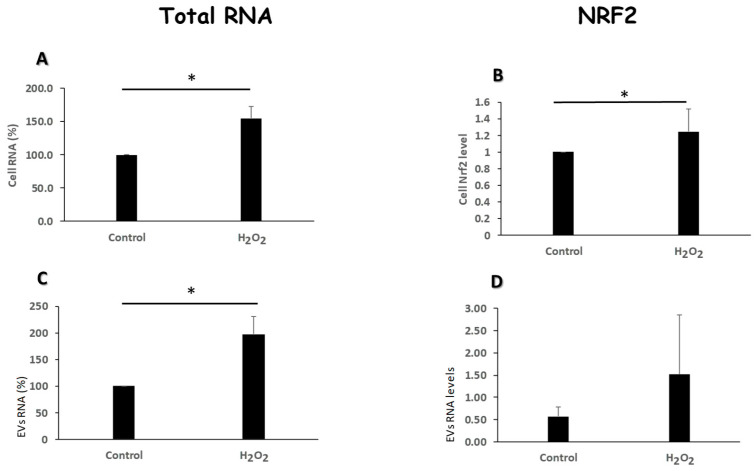
Oxidative stress modulates the amount of RNA content and Nrf2 levels of EVs. K-562 cells were grown in the presence of 75 μM H_2_O_2_ and total RNA was extracted from cells and their cognate EVs. (**A**,**C**). Total RNA was extracted from cells and EVs and measured by the Qubit 2 device. Shown are the normalized values of RNA per number of cells and EVs, respectively. (**B**,**D**). The expression levels of the NRF2 gene transcript was measured by Q-RT-PCR. Shown are the normalized values of RNA per number of cells and EVs, respectively. Each column represents mean ± standard error mean of three independent biologically replicates experiments. * *p* < 0.05, Student *t*-Test.

K-562 cells possess a unique redox balance with a limited capacity to produce ROS. In vivo the redox potential plays a fundamental role in the development of CML and in the resistance to IM [14,15,16]. In light of the relevance of oxidative stress in CML and IM treatment, we focused on exploration of the putative mechanisms of oxidative stress insult on K562 cells and their cognate EVs. RNA was extracted from EVs released into culture media from both H_2_O_2_-treated and untreated cells. As depicted in Figure 4, EVs released from K562 cells grown under oxidative stress conditions were enriched with RNA content compared to those released by untreated cells. Moreover, the RNA content in the mother cells that were cultured under oxidative stress contained more RNA compared to the control untreated cells (Figure 4C).

The *Nrf2* gene product regulates an antioxidant response by activating antioxidant genes and elements [17]. To gain a mechanism related insight, we further studied the expression levels of the *Nrf2* gene in our setting. Q-RT-PCR revealed that the relative expression levels of *Nrf2* did not increase in response to oxidative stress insult either in the mother cells or their cognate EVs (Figure 4B,D).

### 3.5. Increase in ROS Levels Arrest K562 Cells in G2 Phase of the Cell Cycle

**Figure 5:**
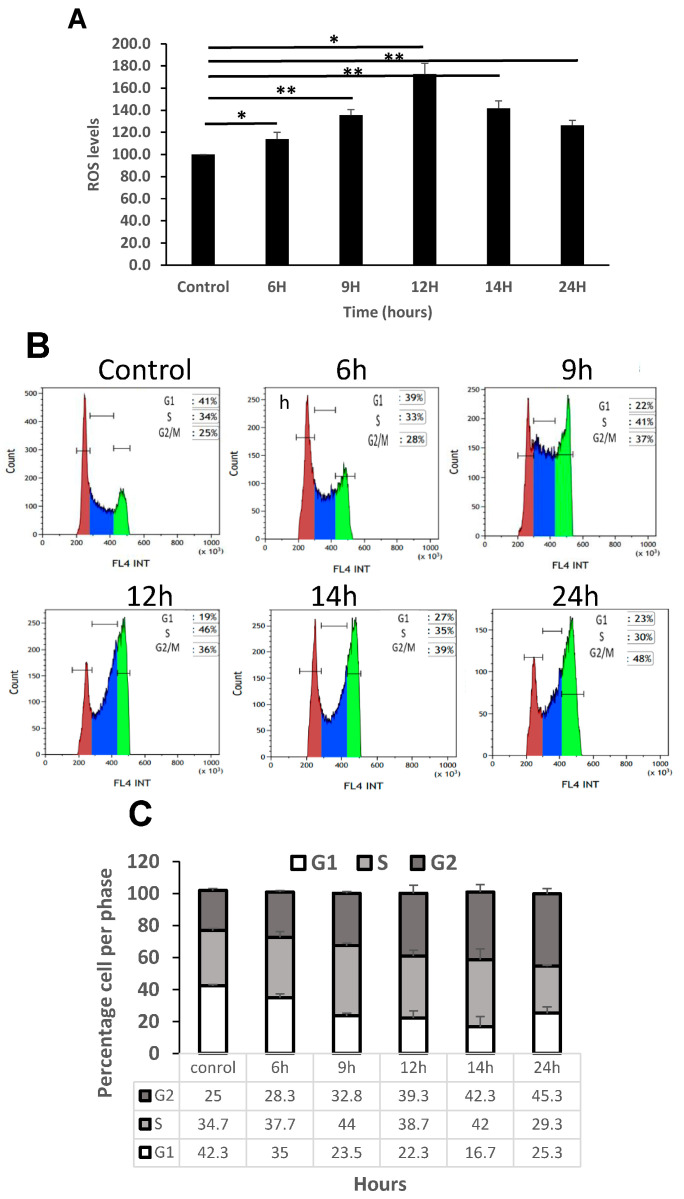
Increase in ROS levels arrest K562 cells in G2 phase of the cell cycle. (**A**). K-562 cells were grown in the presence of 75 μM H_2_O_2_ for several time periods. Intracellular levels of ROS were measured using DC-FHDA by flow cytometry. Each column represents mean ± standard error of three independent experiments. * *p* < 0.05, ** *p* < 0.01, ANOVA and Dunnet. (**B**) A representative example. Time of exposure is indicated above each graph. Cells in G1, S, G2/M are colored in brown, blue and green, respectively. (**C**) Summary of cell cycle analysis of K-562 cells exposed to 75 μM H_2_O_2_ for 24 h. Cells were processed after PI staining and analyzed by flow cytometry. Each column represents mean ± standard error mean of three independent biologically derived replicates experiments.

To verify that external oxidative stress increased the intracellular ROS levels in K562, we measured the fluorescent signal of H2DCFA, an indicator of cellular ROS [18,19], in these cells by flow cytometry. Our results showed that cell treatment with H_2_O_2_ caused a significant increase of the intracellular ROS level during 24 h post exposure, reaching a maximal level at 12 h (about 1.8 fold. Figure 5A).

To study the effect of ROS levels on the cell cycle status in our setting we cultured the cells in the presence of 75 μM H_2_O_2_ for 24 h for different time periods (6, 9, 12, 14 and 24 h) and then stained the cellular DNA cells with propidium iodide. Flow cytometry analysis showed that oxidative stress increased the fragmentation of the DNA and arrested the cell cycle at the S phase, starting after 9 h in K562 cells (Figure 5B,C). As shown in Figure 5B, during 24 h of treatment with H_2_O_2_ the proportion of K562 cells at the G2 phase increased whereas the proportion of cells at the G1/S phase decreased, indicating that oxidative stress probably contributed to G2 phase of the K562 cells. These differences reached only a trend but no statistical significance was obtained in our setting.

### 3.6. ROS Increased Apoptosis and Shrinkage of K562 Cells

**Figure 6:**
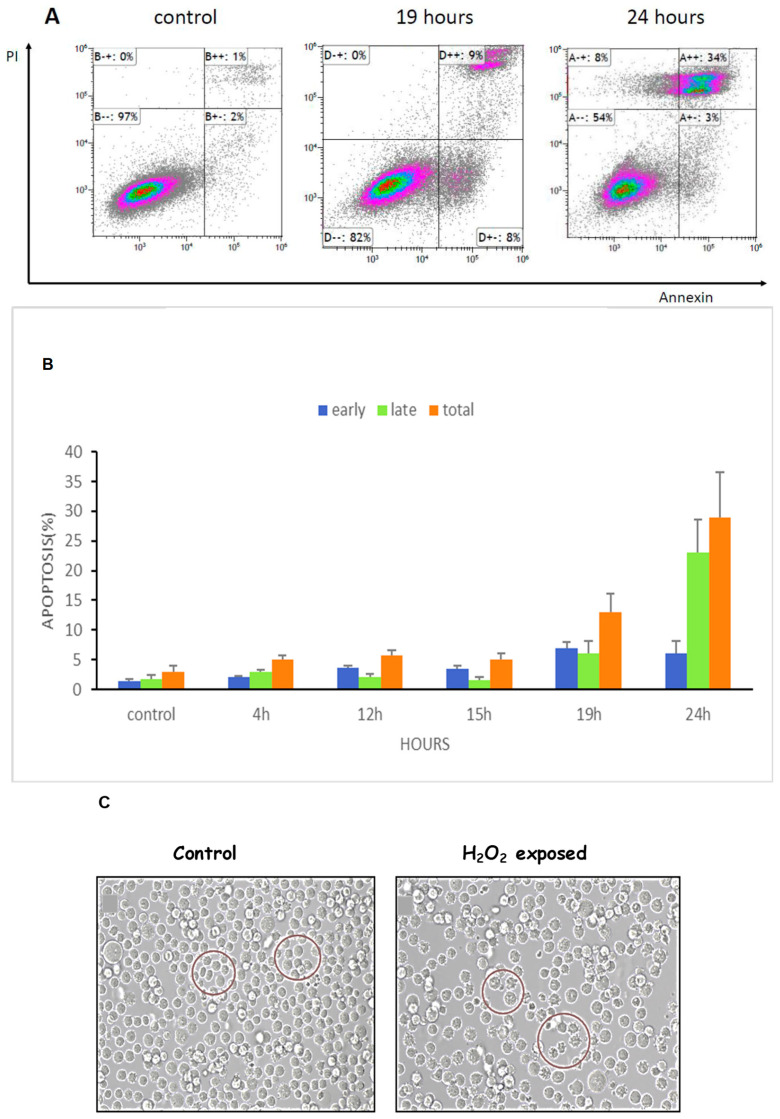
ROS increased apoptosis and shrinkage of K562 cells. Cells were cultured for 24 h in the presence of 75 μM H_2_O_2_. Thereafter, the cells were stained with annexin/PI and analyzed by flow cytometry. (**A**). An example of flow cytometry analysis. (**B**). Quantification of three independent experiments. (**C**). Microscope inspection of the cells post H_2_O_2_ exposure. The circles indicate normal cells in the control group and apoptotic cells in the H_2_O_2_ exposed cells. Each column represents mean ± standard error mean of three biologically independent experiments.

As oxidative stress induced cell cycle arrest and apoptosis in leukemic cells [19], we measured apoptosis in our setting as well in response to elevated ROS levels. For this purpose, cells were exposed to 75 μM of H_2_O_2_, for 4, 12, 15, 19 and 24 h while K562 cells without H_2_O_2_ served as control. Apoptosis was determined using Annexin V and PI staining. As demonstrated in Figure 6A,B, 19 and 24 h post treatment, apoptosis levels increased by 17% and 37%, respectively, relative to that of the untreated control cells.

Apoptosis of cells is manifested, among other features, as cellular shrinking, resulting from condensation and margination of the chromatin and ruffling of the plasma membrane [20]. Therefore, we inspected our treated cells and found that a portion of the culture demonstrated morphologic changes typical of apoptosis (see Figure 6C). Although we did not measure DNA fragmentation in this experiment, we assumed that the shrinkage of the cells was linked to smaller nuclei in which DNA fragmentation occurred. Control untreated cells presented a normal membrane integrity and homogeneous nuclear mass. PI staining and cells shrinkage caused by ROS insult are both typical of the apoptosis process, implying that cells underwent apoptosis in our setting.

### 3.7. EVs Are Taken up by K562 Cells in a Time and a Dose-Dependent Manner

**Figure 7:**
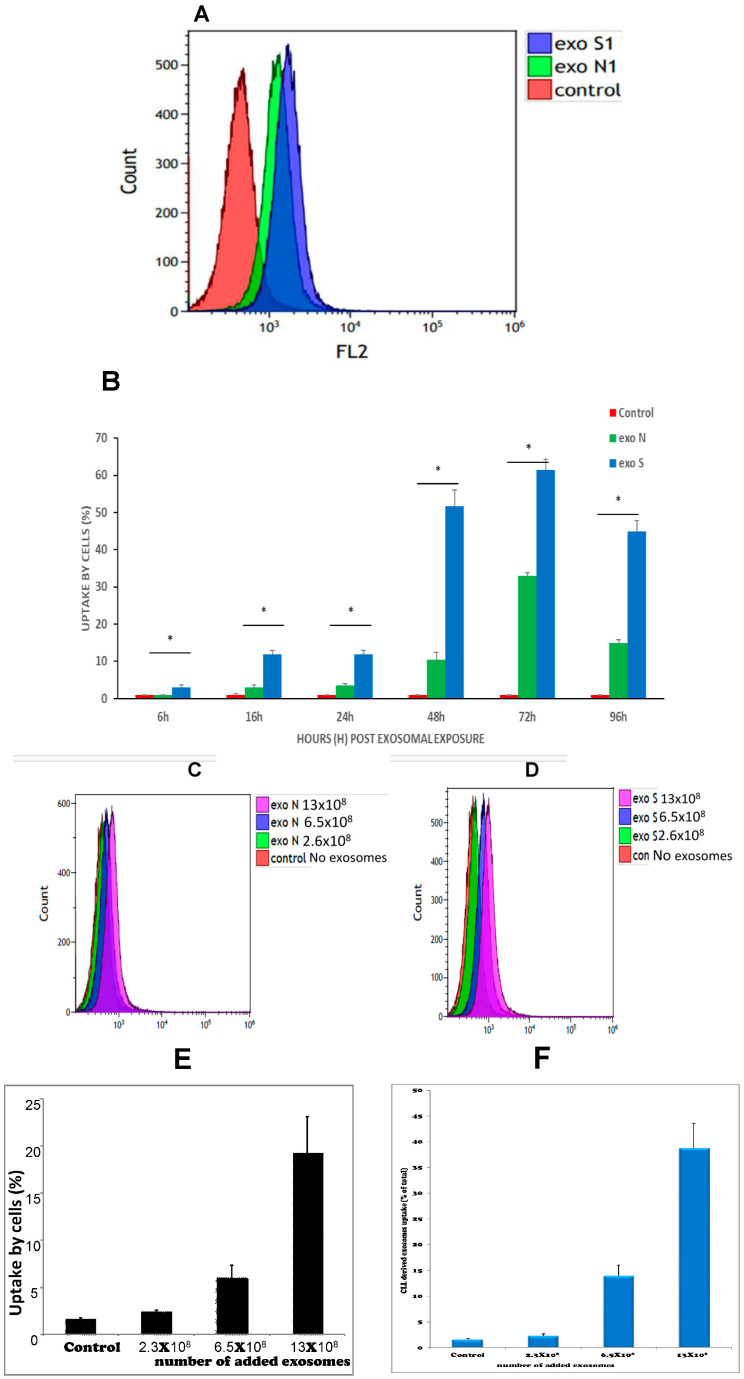
Kinetics (time and dose) of EVs uptake by sensitive K562 (s-K562) cells. Cells were subjected to the stained EVs and the uptake was followed during 96 h by flow cytometry. (**A**). An example of flow cytometry results, analyzing fluorescent FM-1-34 stained EVs by FL-2 channel. EXO denotes EVs. (**B**). Graphic quantitation of three independent experiments. * *p* < 0.05, ANOVA and Dunnet. Cells were exposed to increasing amounts of stained EVs and uptake was measured by flow cytometry. (**C**). A typical flow cytometry analysis of cells exposed to EVs-S collected from the growth media of cells grown in normal conditions (EVs-N) EXO denotes EVs. (**D**). A typical flow cytometry analysis of cells exposed to EVs collected from the growth media of cells grown under oxidative stress conditions (75 μM of H_2_O_2_), exo S. (**E**,**F**). Quantitation of three biologically independent experiments of **C**,**D**. Numbers in (**C**–**F**) refer to the numbers of EVs to which the cells were exposed. Shown are mean ± standard error mean. EXO denotes EVs.

Since we aimed at studying the putative effects of K562-derived EVs on intact K562 cells, we explored the kinetics of EVs engulfment by these cells. EVs were labeled by the FM-1-43 fluorescent membrane probe and the uptake was assessed by flow cytometry. The labeled EVs derived from normal (EVs-N) or oxidative stressed (EVs-S) cells were incubated with intact K562 cells for 6 h, 16 h, 24 h, 48 h and 96 h and K562 cells with PBS only served as control. As depicted in Figure 7A, maximal EVs uptake was demonstrated 48 h and 72 h post exposure. Interestingly, a marked difference of EVs uptake between the two EVs types: those that were secreted from control cells (EVs-N) or from ROS-containing cells (EVs-S) were observed, throughout EVs exposure. The peak difference between these two types, ~40%, was demonstrated 48 h post EVs exposure (Figure 7A). Dose-dependent analysis demonstrated a positive correlation between number of added EVs and the level of engulfment, again with a twofold uptake of oxidative stress-derived EVs (Figure 7B).

These results demonstrate well the ability of K562 cells to take up EVs in a dose and a time-dependent manner.

### 3.8. EVs Derived from Oxidative Stress Exposed Cells (EVs-S) Increase the Proliferation of K562 Cells

**Figure 8:**
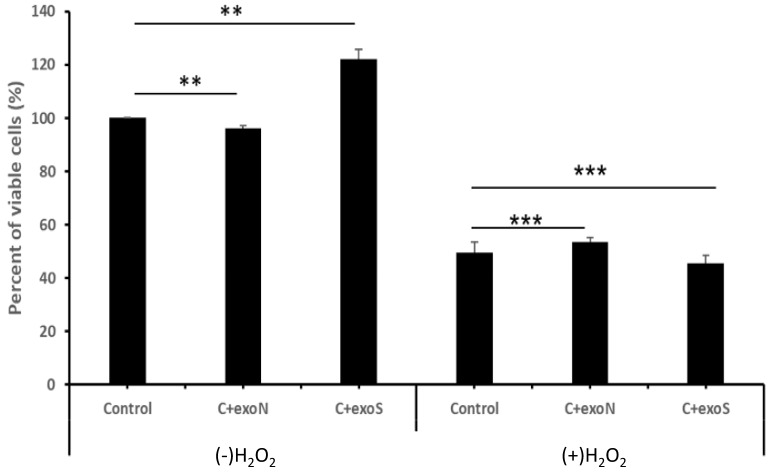
Differential effects of K562-derived EVs derived from cells grown under normal (EVs-N) or oxidative stress (EVs-S) conditions on the proliferation of K562 cells. Cells were exposed to EVs-N or EVs-S and proliferation was assessed by the Trypan blue exclusion assay in response to H_2_O_2_. Left panel: proliferation of cells with no added H_2_O_2_; right panel: proliferation of cells grown under 75 μM of H_2_O_2_. Each bar represents mean and standard error mean of three biologically independent experiments. ** *p* < 0.01, *** *p* < 0.001, ANOVA and Dunnet.

To identify the putative role of EVs secreted from K562 grown under oxidative stress, the two types of K562-derived EVs, EVs-N or EVs-S as above were isolated and incubated with two types of K562 cells for 24 h: either cells that were grown under normal conditions or those that were exposed to 75 μM H_2_O_2_ for 24 h. EVs addition was conducted prior to H_2_O_2_ treatment. As shown in Figure 8, EVs that were secreted in the presence of ROS increased the survival rate of K562 cells grown under normal conditions, compared with the K562 cells incubated with EVs-N EVs, and also compared with K562 cells treated with no EVs. This increase was not detected in cells grown under oxidative stress (Figure 8, right panel). Of note, although the difference in cell viability reached statistical significance, we considered it as biologically negligible. The possible advantage of the increase in proliferation is described in the discussion.

### 3.9. Cells That Are Resistant to IM Secrete Different Number of EVs Compared with IM Sensitive Cells

**Figure 9:**
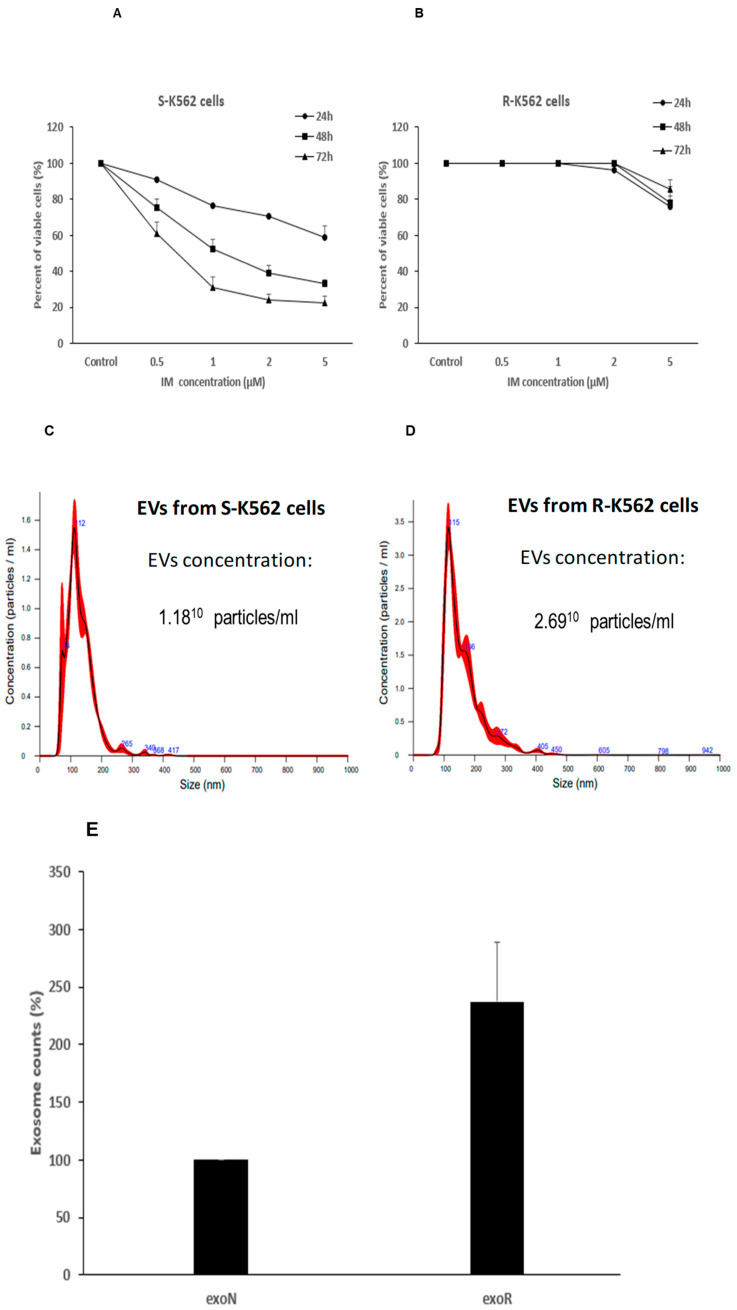
IM differentially affects the viability and number of secreted EVs of IM sensitive vs. IM resistant K562 cells. Cells were grown in the presence of the indicated IM concentrations for 24 h, 48 h and 72 h and their viability was assessed by the WST-1 assay. (**A**). IM sensitive K562 (S-K562). (**B**). IM resistant K-562 cells (R-K562). (**C**,**D**). Nano-sight tracking analysis (NTA) of EVs isolated from IM S-K-562 or IM R-K-562 ((**C**,**D**), respectively), cells. (**E**). R-K-562 secrete more EVs/cells compared to S-K-562 cells. Similar numbers of both cell types were grown for 48 h, EVs were isolated and analyzed by the NTA. Shown is mean and standard error mean of three biologically independent experiments.

At this point of the study we sought to explore the contribution of EVs derived from K562 cells that are resistant to IM to the sensitivity of intact K562 cells, in the context of oxidative stress. We used two types of cells: those that are sensitive to IM (same cells which we used so far), termed S-K562, and cells that are resistant to the drug, which we termed R-K562. Firstly, we exposed the two cell types to a range of IM concentration and measured viability thereafter. As expected, 24, 48 and 72 h post IM treatment the IC50 of the cells was about 5 μM, 1 μM and 0.5 μM, respectively, whereas the resistant cells showed 100% viability under these IM concentrations. Viability was measured both by the Trypan blue exclusion and the WST-1 assays. Since the results of both methods were similar, we show here only the results of the WST-1 assay (Figure 9A,B). After validating IM resistance of the R-K562 cells we proceeded to assess the number of EVs secreted by each cell type. S-K562 and R-K562 cells were cultured with EVs-free medium and their secreted EVs were analyzed by NTA. Interestingly, the number of EVs isolated from the resistant cells was ~2 times higher compared with that of the sensitive S-K562 cells (Figure 9C).

### 3.10. K562-R EVs Confer Partial Protection from Oxidative Stress and IM

Generally, EVs exert phenotypic effects in recipient cells through their engulfment by the cells and the subsequent release of their cargo. To identify whether R-K562 and S-K562 EVs can modulate IM-resistance in K562 cells, we collected EVs from S-K562 exposed to H_2_O_2_ or control EVs without H_2_O_2_ stimulation (EVs-S, EVs-N respectively). Thereafter, R-K562 and S-K562 cells were incubated with these EVs for 24 h, prior to the addition of 1 and 2 μM IM for 1 and 2 days. Viability of the exposed cells was measured by WST-1 assay.

**Figure 10:**
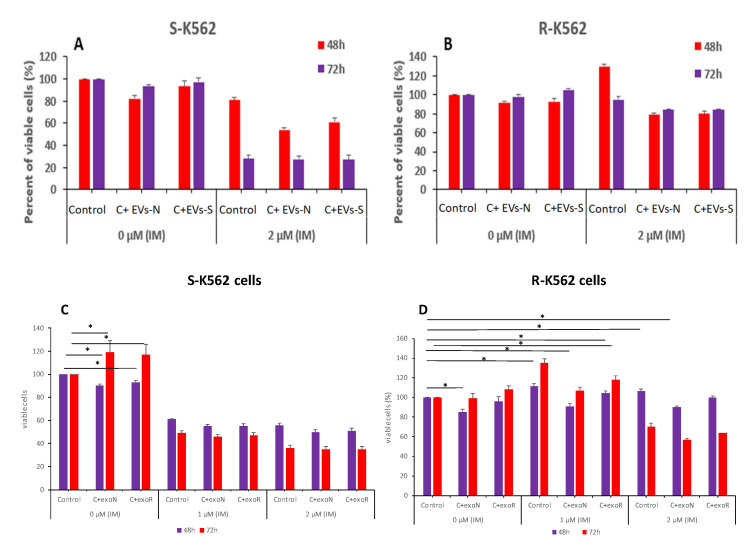
IM and oxidative stress-derived EVs differentially affect the viability of K562 cells (upper panel). (**A**) S-K-562 and (**B**) R-K562 cells were grown with no EVs (control) or after treatment with EVs derived from either cells grown in normal (EVs-N) conditions or those that derived from cells grown under oxidative stress conditions (EVs-S) for 48 h and 72 h. At the end of cell incubation with EVs, 2 μM IM was added for an additional 24 h and cell viability was assessed by the WST-1 assay. Shown is mean and standard error mean of three biologically independent experiments. IM and EVs derived from IM sensitive or resistant cells differentially affect the viability of K562 cells (lower panel). (**C**) S-K-562 and (**D**) R-K562 cells were grown with no EVs (control) or after treatment with EVs derived from either sensitive (S-K-562, EVs-N) cells or from IM-resistant (R-K-562, EVs-R) cells for 48 h and 72 h. At the end of cells incubation with EVs, IM was added for additional 24 h and cell viability was assessed by the WST-1 assay. Shown is the mean and standard error mean of three biologically independent experiments. * *p* < 0.05.

All in all, the results of this set of experiments showed the following:

- The S-K562 EVs-treated (EVs-N and EVs-S) cells presented a decrease in cell proliferation (Figure 10A), compared with S-K562 cells without EVs treatment (control), suggesting that S-K562 cell-derived EVs do not confer drug resistance phenotype to S-K562 cells.
- EVs from S-K562 cells (EVs-N and EVs-S) decreased cell viability of R-K562 cells in the presence of IM as shown in Figure 10B compared to control non-EVs exposed cells, although this did not reach statistically significance.
- EVs from R-K562 cells (EVs-R) increased the survival of R-K562 cells in the presence of 1, 2 μM IM compared to EVs from S-K562 (EVs-N), as shown in Figure 10D.

We concluded that the specific composition of R-K562-derived EVs mediated the enhanced survival of exposed S-K562 cells.

## 4. Discussion

Although the treatment and prognosis of CML have markedly improved since the development of TKIs, not all patients with CML respond well. During the progression of leukemia, exosomes or EVs secreted by the neoplastic cells may increase the aggressiveness of the disease by upregulating the proliferation of the leukemic cells, promoting angiogenesis and inhibiting hematopoiesis. Additionally, EVs are involved in providing resistance to drugs in general and to anti-cancer drugs in particular [21,22,23,24].

In light of the need to clarify the mechanisms contributing to development of resistance to TKI’s on the one hand and the altered metabolism of oxidative stress imposed by IM on the other hand, we designed our study focusing on the putative role of oxidative stress in conferring resistant to the drug through EVs shuttling. EVs shuttling was chosen based on several studies showing their role in cellular response to oxidative stress [25,26,27].

As expected, our study demonstrated that ROS (imposed by H_2_O_2_) may exert significant inhibition of cell proliferation in a dose- and a time-dependent manner. Interestingly, the surviving cells released more EVs per cell. Similarly, proliferative stress induced by decreasing the concentrations of FBS in the growth media of the cells also induced decreased cellular proliferation and increased the number of EVs secretion per cell. These cellular responses are in line with previous studies showing that the quality and quantity of extracellular vesicles vary depending on the physiological status of the mother cells [26], which stems from environmental stresses such as oxidative stress and heat [27,28,29].

Interestingly, EVs released under stress conditions differed in their RNA levels compared to those released from cells growing under normal conditions. Oxidative stress induced the engulfment of bigger amount of RNA in the exposed cells’ EVs. Differential protein and RNA content in EVs released from cells exposed to stress conditions has previously been reported [30].

The *Nrf2* gene product is implicated in protecting cells from oxidative stress [31]. Whereas the exposed cells increased the levels of the *Nrf2* gene expression in response to oxidative insult, their secreted EVs did not demonstrate a similar increase. This difference serves as an example for the active engulfment of molecules into EVs; in this case, the presence of the gene transcripts in the cells but not in their cognate EVs. Our results suggest that at least on the transcriptional level, *Nrf2* is probably not involved in conferring any protection from oxidative stress to other K562 cells in our setting. This result contrasts with other studies [31], demonstrating an increase of the expression of the *Nrf2* gene in response to oxidative insult in cells and their released EVs. As the mechanism of molecular packaging into EVs is far from being elucidated, it may also vary among different cellular contexts.

Oxidative exposure promoted morphological changes typical of apoptotic cell death of our cells, as previously reported [32,33]. Apoptosis was shown to be the result of the accumulation of ROS which damaged DNA, lipids and proteins. In agreement with this, our data showed that under environmental stress, ROS levels increased markedly, probably causing damage to cell structure and finally inducing apoptosis. Moreover, we detected a dose-dependent arrest in the G2 phase of K562 cell cycle by H_2_O_2_. The result of this analysis suggests that inducing apoptosis and cell cycle arrest may be a key mechanism by which H_2_O_2_ inhibits s-K562 cell proliferation.

Another effect of oxidative stress was observed while assessing EVs uptake by intact K562 cells. Furthermore, s-K562-derived EVs growing under oxidative stress (EVs-S) were taken up rapidly and preferentially compared to EVs secreted from the control cells growing under normal conditions (EVs-N). Along these lines, Mutschelknaus et al. reported that radiation increases EVs release and uptake in head and neck squamous carcinoma cells, although the underlying process remained unclear [34]. Another study showed that the uptake of EVs by hepatocellular carcinoma cells was much more efficient than the uptake of the same EVs by normal liver cells [35].

To elucidate the putative protective effects of our EVs on oxidative stress-exposed cells we have measured the proliferation of these cells in our setting under these conditions. We provide evidence that s-K562-derived EVs exposed to oxidative stress increased the proliferation of intact s-K562 cells; however, when the intact recipient K562 cells were exposed to oxidative stress this effect was not obtained. Previous studies have reported that CML EVs trigger an anti-apoptotic phenotype in the recipient cells. These studies suggested that the secretion of EVs by CML cells could have potentially contributed to the progression of leukemia through triggering of an autocrine loop, thus representing a possible target for new therapies [36,37,38]. Our results may add another feature regarding the role of EVs-S in CML progression.

IM, the first TKI treatment for CML, is implicated in changes of the neoplastic cells’ oxidative environment, which may impose resistance to the treated cells [39]. Assessing the effects of H_2_O_2_-derived EVs on the response to IM-exposed sensitive and resistant cells, revealed several interesting findings.

Apart from the differential sensitivity to IM, the number of EVs secreted by these cells presented a major difference between the two cell types, (s-K562 and r-K562). The r-K562 cells secreted twice as many EVs compared to those secreted by s-K562 cells, as reported also in a recently published study [29].

The s-K562 cell-derived EVs did not confer a drug resistance phenotype to the s-K562 cells, as incubation of these cells with EVs originating either from r-K562 or s-K562 prior to IM did not provide any protection from the drug and even decreased s-K562 cell proliferation in response to 48 h incubation with IM.

In contrast, EVs originating from r-K562 cells (EVs-R) increased the resistance of r-K562 cells to IM compared to those secreted from s-K562 cells (EVs-N), indicating that they may provide IM resistance at least in our setting.

Of note, it is possible that resistance greater than that observed in the presence of extracellular vesicles from BCR-ABL1-expressing cells with imatinib resistance-associated mutations, such as T315I, could occur. Additionally, it is also possible that resistance greater than that observed in the presence of extracellular vesicles from cells expressing other BCR-ABL1 variants could arise.

Numerous studies demonstrated that exposure to IM increased the level of DNA damage in susceptible K562 cells compared to that of resistant K562 cells. While undergoing this process, IM-susceptible cells accumulated additional DNA damage which, in turn, was one of the factors contributing to rapid cell death [15,40].

Previous studies have also reported that CML cells can release EVs, and that they can be transferred between CML cells. Upon engulfment and subsequent release of their cargo, they exert phenotypic changes in the recipient cells [11,12,36,41].

EVs derived from r-K562 cells had no measurable effects on the survival rate of s-K562 cells in the presence of IM. Interestingly, exosomes from imatinib-resistant CML cells can differentially affect sensitive cells due to variations in their molecular cargo, release mechanisms and interactions with the tumor microenvironment. Key explanations supported by research include the following: 1. Protein and miRNA cargo differences: specific exosomal proteins (e.g., IFITM3, CD146, CD36) and miRNAs (e.g., miR-365) may be transferred to sensitive cells, enhancing survival and reducing apoptosis [21,23,24]. RPL13 and RPL14, both are upregulated in plasma exosomes from imatinib-resistant patients are linked to ribosomal protein synthesis, which may counteract drug effects [42]. Other recent studies have shown that exosomes derived from drug-resistant cancer cells transmit chemoresistance by the transfer of extracellular vesicles containing miRNAs in breast cancer cells [43]. 2. The effect of autophagy-dependent exosome release: imatinib-resistant cells exhibit increased autophagy activity, promoting exosome release. Drugs like dasatinib reduce exosomal secretion by downregulating autophagy proteins (e.g., beclin-1, Vps34), indirectly influencing recipient cell responses [44]. 3. Stromal cell-derived exosomes from bone marrow mesenchymal stem cells MSCs or macrophages can paradoxically promote drug resistance in vivo by delivering miRNAs (e.g., miR-21) or activating pathways like NOTCH3, even if they inhibit proliferation in vitro [45]. 4. Microenvironmental context: in vivo, exosomes interact with stromal cells, endothelial cells, or immune cells in the bone marrow, creating a protective niche that enhances resistance. This contrasts with simpler in vitro models, where such interactions are absent [46]. 5. Engineered exosome therapy studies revealed that modifying exosomal content can reverse resistance, highlighting the importance of cargo specificity [47].

The discrepancy between these reports and our results may stem from different EVs treatments administered in each study. Although we did increase the number of EVs applied to the cells, we may have missed the optimal EVs amount that might have provided cellular protection from IM. Another possible explanation may be related to differential roles of EVs-mediated survival among patients with CML. In this regard, the mechanism and characteristics of IM resistance may vary among patients and cell lines, thus explaining discrepancies between our study and the above-mentioned ones. IM resistance may manifest with different phenotypes, cellular proliferation rates and other cellular properties, including EVs content and the rate of EVs shedding.

Interestingly, EVs originating from s-K562 cells grown with no oxidative stress (EVs-N) decreased cell viability of r-K562 cells in the presence or absence of IM. Further studies should determine whether this phenomenon will be important in the context of the development of resistance to the drug in patients with CML.

We demonstrated that after incubation with EVs derived from s-K562, a decrease in the survival of the r-K562 cells in both conditions with toxic doses of 1 μM IM occurred. With normal conditions, on the other hand, EVs derived from r-K562 further increased the proliferation of the r-K562 cells. Although we do not have an explanation for this phenomenon, two recent studies may clarify this point [48,49]. Wuxiao et al. reported that exosomal micro-RNA 145, secreted from K562 cells, decreased the proliferation of intact K562 cells exposed to adriamycin by inhibiting ATP-binding cassette sub-family E member 1 (ABCE1). These findings demonstrate that the overexpression of miR 145 promoted leukemic cell apoptosis and enhances the sensitivity of K562/ADM cells to ADM by inhibiting ABCE143 [49]. The results of our study showed that the protection from oxidative stress provided by K-562 cell-derived EVs exposed to oxidative stress is only partial. However, these EVs can provide intact K-562 cells with some resistance to IM treatment. This result suggests that the resistance to IM may be developed and expand to other cells by EVs that are secreted from already resistant cells, similarly to a horizontal transfer of resistance provided by plasmids in bacteria [50].

Our study suffers from several limitations. Firstly, the lack of specific techniques, such as protease treatment, to definitively distinguish between EVs that may have attached the K-562 cell membrane and those EVs that were internalized into the recipient cells. The scientific literature presents conflicting approaches to this challenge. While some researchers advocate for protease treatment [51], others argue that such enzymatic treatment can disrupt natural uptake mechanisms, cause cellular membrane damage and create experimental artifacts (e.g., [52]). Importantly, the vast majority of studies do not use any protease inhibitor prior to the analysis of EVs uptake. In our study, however, we observed clear functional effects within the cells, which are unlikely to result from mere surface binding but point to EVs internalization. These include the differential effects of EVs derived from K562 cells grown under normal (EVs-N) or oxidative stress (EVs-S) conditions on cellular proliferation (Figure 8, Figure 10). However, having said that, we cannot rule out effects of non-vesicular components or surface-bound EVs that may have been present during EVs isolation, triggering signaling in our setting.

Other drawbacks will be addressed in a subsequent study currently being conducted in our laboratory. These include the following: 1. No molecular analyses of our EVs content was performed. 2. We have not analyzed the effect of EVs isolated from CML patients in our setting. 3. No experiment of blocking the secretion of EVs was conducted as a negative control. 4. We did not study the presence of receptors on K562 membrane that can selectively take up stressed EVs versus normal EVs. 5. The EVs effects on third generations of TKIs such as dasatinib, nilotinib or ponatinib were not tested. 6. We do not yet know how stable the resistant phenotype is over time, but, as mentioned above, this point will be checked by us. 7. No experiments with antioxidants or activators of *nrf2* were conducted as another type of control. 8. The dose–response relationship between EVs concentration and the magnitude of imatinib resistance was executed. 9. We did not study the possible differences between genomic or mitochondrial DNA in exosomes derived from either imatinib resistant or sensitive ones that may have contributed to horizontal gene transfer into the recipient cells. 10. Analyses of the molecular cargo of the various EVs in our setting including proteome and gene expression was not conducted in the current study.

The clinical significance of our reported findings should await the results of the above-described expanded future studies and may also include an interference of EVs secretion in patients with CML.

## Data Availability

Data are contained within the article.

## Abbreviations

The following abbreviations are used in this manuscript:

CML	Chronic Myeloid Leukemia
S-K562	Sensitive Cell Line
R-K562	Resistant Cell Line
MtDNA	Mitochondrial DNA
ROS	Reactive Oxygen Species
EVs	Extracellular Vesicles
IM	Imatinib Mesylate
TKIs	Tyrosine Kinase Inhibitor
FBS	Fetal Bovine Serum
Nrf2	The nuclear factor erythroid 2—related factor 2
